# Multi-Omics Analysis Provides New Insights into the Interplay Between Gut Microbiota, Fatty Acid Metabolism, and Immune Response in Cultured and Wild *Coilia nasus* from the Yangtze River Area in China

**DOI:** 10.3390/microorganisms13071711

**Published:** 2025-07-21

**Authors:** Chang Yang, Kai Liu, Yanmin Deng, Qianhui Wang, Shiqian Cao, Qunlan Zhou

**Affiliations:** 1 Wuxi Fisheries College, Nanjing Agricultural University, Wuxi 214128, China; 2021113023@stu.njau.edu.cn (C.Y.); liuk@ffrc.cn (K.L.); dengym530@163.com (Y.D.); 2024113023@stu.njau.edu.cn (S.C.); 2 Freshwater Fisheries Research Center, Chinese Academy of Fishery Science, Wuxi 214081, China; 3 School of Fisheries and Life Science, Dalian Ocean University, Dalian 116023, China; 17690505665@163.com

**Keywords:** estuarine tapertail anchovy, polyunsaturated fatty acid, immunity, muscle transcriptome, 16S rRNA sequencing

## Abstract

To elucidate the interactions among fatty acid metabolism, immune status, and gut microbiota, both cultured and wild *Coilia nasus* from the Yangtze River were examined in China. The results demonstrated that wild *C. nasus* exhibited markedly higher lipid and docosahexaenoic acid (DHA) contents, a greater ratio of total ω-3 PUFAs to total ω-6 PUFAs, and more active antioxidant enzymes compared to cultured *C. nasus*. However, the shear force, water-holding capacity, and total n-6 PUFA content were lower in wild *C. nasus*. Transcriptome analysis revealed distinct gene expression patterns: wild *C. nasus* upregulated immune-related genes, while cultured *C. nasus* downregulated genes related to fatty acid metabolism. Significant differences were observed in alpha and beta diversity between cultured and wild groups. LEfSe analysis identified *Clostridium_T*, *Escherichia*, and *Glutamicibacter* as biomarkers for cultured *C. nasus*, while eight genera, including *Pseudomonas_E* and *Sphingomonas_L*, were predominant in wild *C. nasus*. Modular analysis identified five modules linked to immune functions and fatty acid metabolism. *Clostridium_T*, *Sphingomonas_L*, and *Pseudomonas_E* were dominant in the first two modules, with *Pseudomonas_E* and *Clostridium_T* as key regulators of fatty acid metabolism and immune processes. These differences, likely due to gut microbiota variations, provide insights for *C. nasus* nutritional studies.

## 1. Introduction

Fish is a vital source of long-chain (≥C20) polyunsaturated fatty acids (LC-PUFAs), specifically eicosapentaenoic acid (EPA) and docosahexaenoic acid (DHA). The levels of n-3 LC-PUFA fluctuate due to factors such as species, size, seasonality, and dietary composition. Oily fish, like salmon (*Salmo salar*) and herring (*Clupea harengus*), are particularly rich in both lipids and n-3 LC-PUFA [1]. In aquaculture, the n-3 LC-PUFA content in fish is obviously influenced by their feed. Compared to wild fish, the fatty acid profile of farmed fish muscle seems to be less favorable. But the further mechanism still needs to be clarified.

The gut microbiota, often conceptualized as a virtual organ, is crucial in host metabolism and physiological functions [2], particularly in metabolic processes and immunomodulation [3,4]. Although muscles are anatomically distant from the gut, the observed correlation between alterations in gut microbiota composition, compromised physiological states, and muscle catabolism suggests that the microbiota may exert a direct or indirect influence on muscle mass and regulation [5,6]. Signals generated from gut–microbiome interactions, such as microbial metabolites, peptides, lipopolysaccharides, and interleukins, act as intermediaries linking gut microbiota activity to muscle function. These signals modulate muscle function by influencing systemic and tissue-specific inflammation as well as insulin sensitivity [7]. One potential mechanism is that the gut microbiota may be involved in regulating muscle sensitivity to anabolic signals [5,8]. By utilizing advanced technologies to predict the functions of specific bacterial communities, it may be possible to optimize the nutritional profile and immune status of fish muscles.

With advancements in molecular biology and bioinformatics, multi-omics approaches, including genomics, transcriptomics, proteomics, metabolomics, and microbiomics, have become crucial for thoroughly exploring aquatic biological processes and complex biological systems. Multi-omics analyses have elucidated novel metabolic pathways in fish, which are crucial for understanding how fish adapt to their environments and maintain survival [9]. In addition, they have played a crucial role in elucidating the underlying causes of fish diseases, providing robust support for the development of appropriate treatment strategies and enhancing the understanding of pathogenesis [10]. An investigation into the gut microbiota and plasma metabolome revealed that cultured Eurasian perch (*Perca fluviatilis*) exhibit lower protein digestibility but higher plant polysaccharide digestibility compared to their wild counterparts [11]. A substantial body of research indicates that multi-omics approaches can reveal a wider array of mechanisms within the domains of fish toxicology, pharmacology, physiology, and pathology.

The estuarine tapertail anchovy (*Coilia nasus*), a coastal anadromous fish classified under the Clupeiformes order and Engraulidae family [12], demonstrates a wide distribution along China’s East China Sea and Yellow Sea coastal regions, as well as in major rivers including the Yangtze, Yellow, and Qiantang [13]. Esteemed for its high fat content, delicate flesh, and unique flavor, this species is celebrated as one of the “Three Delicacies of the Yangtze River.” Prior to the 2000s, *C. nasus* was abundant in the Yangtze River ecosystem and was recognized as the region’s most economically vital fishery resource. However, excessive fishing pressures led to its decline, prompting authorities to implement a commercial fishing moratorium on wild populations to facilitate recovery. Currently, preliminary signs of resource replenishment are observable. Concurrently, the aquaculture industry focusing on *C. nasus* has undergone substantial expansion; however, cultured specimens consistently demonstrate a lower nutritional profile compared to their wild counterparts. The artificial feeds used in aquaculture systems provide a variety of nutrients that affect not only growth patterns but also the composition of muscle tissue, particularly influencing the quantity and quality of lipids [14]. Consequently, it is imperative to employ multi-omics technologies to investigate the interrelationships among gut microbiota, fatty acid metabolism, and immune processes in both wild and cultured *C. nasus* to establish a theoretical foundation for the nutritional research of *C. nasus* and the formulation of artificial feeds, ultimately enhancing the nutritional value of cultured *C. nasus*.

## 2. Materials and Methods

### 2.1. Sample Collection

**Figure 1:**
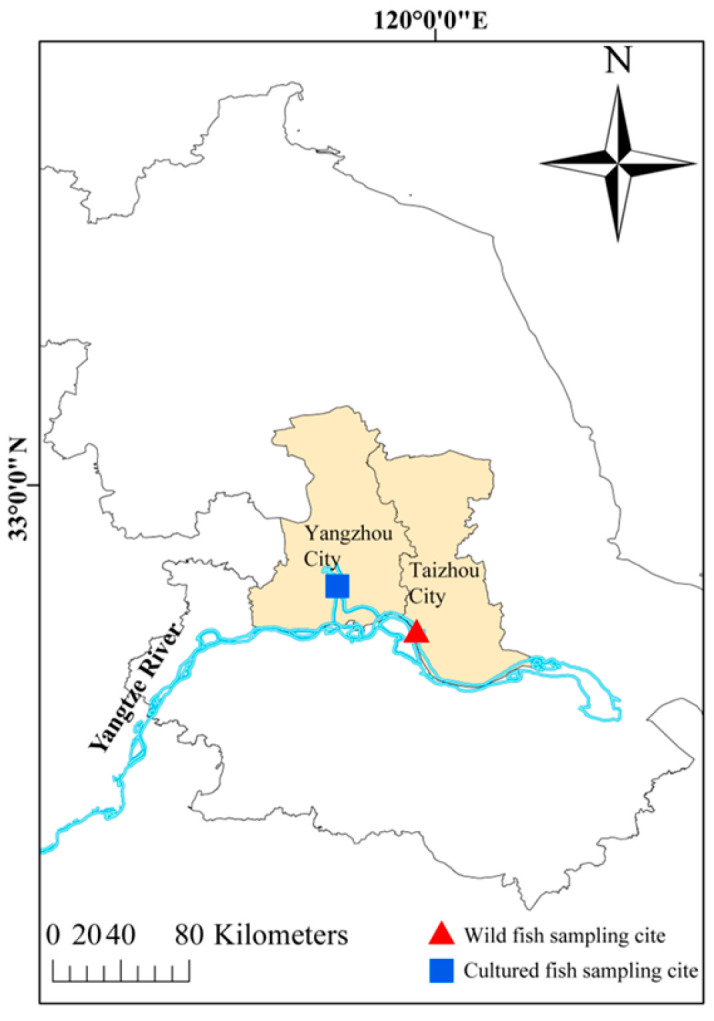
Sampling locations of cultured and wild *C. nasus*.

Between June and November 2023, ten (10) *C. nasus* were collected from two distinct locations in or near the Yangtze River (119.54 E 32.28 N, 119.29 E 32.14 N) in Jiangsu Province, China, to investigate variations in muscle physical and chemical properties, as well as gut microbiota composition, between cultured and wild populations (Figure 1). Specifically, five (5) wild *C. nasus* samples, with an average body length of 317.46 ± 5.23 mm, were obtained from the Taizhou section in the Yangtze River, while the other five (5) cultured specimens, averaging 193.73 ± 5.47 mm in body length, were sourced from an intensive pond culture farm in Yangzhou.

The collection process adhered to ethical guidelines, ensuring the humane treatment of the fish. The specimens were anesthetized using ice water prior to dissection, and then samples of gut and dorsal muscle were extracted. These muscle samples were vacuum-sealed and transported on ice to the laboratory, where they were divided into six sections for analyses of proximate composition, amino acids, fatty acids, texture, water-holding capacity, and transcriptome. Meanwhile, gut samples from each fish were collected and preserved at −80 °C for subsequent processing.

### 2.2. Determination of Muscle Chemical Composition

#### 2.2.1. Proximate Composition Measurement

The proximate composition of the muscle samples was measured using the AOAC standard methods [15]. To assess moisture content, samples were placed in a drying oven at 105 °C until a constant weight was achieved. The crude protein content was quantified using a semi-automatic Kjeldahl apparatus after acid digestion of the dried sample, applying the conversion factor N × 6.25. The crude lipid content was estimated via the ether extraction method. For ash content determination, the dried samples were placed in a muffle furnace at 550 °C for approximately 5 h.

#### 2.2.2. Amino Acid Content Analysis

The amino acid profile was analyzed following the method described by Muhammad et al. [16]. About 0.1 g of the sample was hydrolyzed with 5 mL of 6 mol/L hydrochloric acid at 130 °C for 7 h. The resulting hydrolysate was diluted to 100 mL with distilled water. A 1 mL aliquot of the hydrolysate was freeze-dried and reconstituted in 1 mL of 0.02 mol/L HCl. The amino acid profile was detected with an automatic amino acid analyzer (Hitachi L-8900, Tokyo, Japan) following filtration of the solution through a 0.22 µm pore membrane.

#### 2.2.3. Fatty Acid Content Analysis

The fatty acid profile was detected following the procedure reported by Gladyshev et al. [17]. Lipids were extracted from samples using a mixture of chloroform and methanol with a ratio of 2:1 by volume. The isolated lipids were subjected to hydrolysis via reflux in a methanolic sodium hydroxide solution (8 mg/mL) for 10 min. Subsequently, the mixture was treated with an excess of 3% sulfuric acid in methanol and then subjected to reflux at 90 °C for an additional 10 min to facilitate the synthesis of fatty acid methyl esters (FAMEs). After washing twice with a saturated NaCl solution, FAMEs were extracted with hexane. Finally, the FAMEs were detected using a Network GC System (6890N, Agilent Technologies Inc., Santa Clara, CA, USA), which featured a 100 m long capillary HP-FFAP column (CD-2560, 100 m × 0.25 mm × 0.2 μm, Woogood, Beijing, China). The standard solution with 37 FAMEs Mix (TMRM Quality Inspection Technology, Changzhou, China, Batch G25040153) was employed to set up a standard curve, with 5 different points from 5 mg/mL to 35 mg/mL. Qualitative identification was achieved through retention time matching and peak order verification against the standard. Quantitative analysis employed the standard curve with methyl undecanoate (C11:0 ME) as an internal standard to ensure precision (RSD < 5%).

#### 2.2.4. Antioxidant Capacity Assay

Approximately 0.1 g of fish muscle was homogenized in 0.9 mL of 0.85% saline and then centrifuged at 4 °C and 1500× *g* for 10 min to obtain the supernatant. Protein concentration was measured using the Coomassie brilliant blue method with a kit from Nanjing Jiancheng Bioengineering Institute (Nanjing, China). Malondialdehyde content (MDA), and the activities of superoxide dismutase (SOD), catalase (CAT), and total antioxidant capacity (T-AOC) were also assessed using their assay kits.

### 2.3. Determination of Muscle Physicochemical Properties

#### 2.3.1. Texture Analysis

A TA-XT plusC texture analyzer (Stable Micro Systems, Godalming, Surrey GU8 1YL, UK) was employed to evaluate various texture characteristics, including hardness, springiness, cohesiveness, gumminess, chewiness, resilience, and shear force. A cylindrical probe (P/36R) was equipped, and both the force capacity and the load cell were rated at 500 N. For the texture profile analysis (TPA) test, samples measuring 2 cm × 2 cm × 2 cm were extracted from the muscle. The probe was lowered onto the sample at a speed of 1 mm/s until it compressed the sample to 60% of its original height. Each TPA test involved a single compression cycle of the sample.

#### 2.3.2. Water-Holding Capacity Measurement

To minimize the variability in water-holding capacity (WHC), a standardized sample of 1.20 g muscle tissue was collected. WHC was evaluated by measuring both drip loss and cooking loss. For the determination of drip loss, a fresh sample of back muscle from the same side was weighed, placed in a centrifuge tube lined with blotting paper to absorb moisture, and centrifuged at 4 °C and 2000× *g* for 20 min. Following centrifugation, any remaining moisture was removed using blotting paper, and the muscle sample was reweighed.

Drip loss was determined using the following formula:

Muscle drip loss = 100 × (W_0_ − W_t_)/W_0_

where W_0_ denotes the muscle mass prior to centrifugation and W_t_ is the muscle mass following centrifugation.

For the assessment of cooking loss, a fresh back muscle sample was gathered, weighed, and blotted dry to eliminate residual water and blood. The muscle was then sealed in a bag and subjected to heat treatment at 70 °C for 15 min. After cooling, the muscle was dried with blotting paper and reweighed.

Cooking loss was calculated using the following formula:

Muscle cooking loss = 100 × (W_0_ − W_t_)/W_0_

where W_0_ is the muscle mass before cooking and W_t_ is the mass after cooking.

### 2.4. Muscle Transcriptome Analysis

RNA was extracted from muscle samples using TRIzol (Thermo Fisher, Waltham, MA, USA). Concentration and purity were checked with a Nanodrop spectrophotometer (Thermo Fisher Scientific Inc., Waltham, MA, USA), and integrity was verified by agarose gel electrophoresis. Samples with at least 1 µg of RNA underwent cDNA synthesis via the NEBNext Ultra II RNA Library Prep Kit (New England Biolabs, Ipswich, MA, USA). The resultant products were purified with the AMPure XP system (Beckman Coulter Inc., Brea, CA, USA) and quantified employing the Agilent high-sensitivity DNA assay on a Bioanalyzer 2100 (Agilent Technologies Inc., Newark, DE, USA). Subsequently, the sequencing library was processed on the NovaSeq 6000 platform (Illumina, Shanghai Personal Biotechnology Co., Ltd., Shanghai, China).

Post-sequencing, the data underwent quality filtering with FASTQ (v0.22.0) and were aligned to the *C. nasus* reference genome (accessible at https://gigadb.org/dataset/100677, accessed on 13 September 2024) via HISAT2 (v2.1.0). Transcriptome assembly and merging were carried out with StringTie (v2.1.6) and Gffcompare (v0.9.8). Gene expression was quantified using StringTie and Ballgown, with FPKM values. DESeq2 (v1.38.3) was used for differential gene expression (DEG) analysis, identifying DEGs with |log_2_FoldChange| >1 and *p*-value < 0.05. Enrichment analyses of the DEGs for Gene Ontology (GO) terms and KEGG pathways were conducted using ClusterProfiler (v4.6.0).

### 2.5. Gut Microbiome Analysis

Microbial DNA was extracted directly from the gut employing the E.Z.N.A.^®^ Soil DNA Kit (Omega Bio-tek Inc., Norcross, GA, USA). The V3-V4 region of the microbial 16S rRNA gene was PCR-amplified with universal primers 341F (5′-CCTAYGGGRBGCASCAG-3′) and 806R (5′-GGACTACNNGGGTATCTAAT-3′). Following quantification, pooling, and purification of the PCR products, the purified library was sequenced on the NovaSeq 6000 platform (Illumina, San Diego, CA, USA).

Clean reads were generated for subsequent analysis by merging raw sequencing data using FLASH (version 1.2.11) [18]. Chimeric sequences were identified and removed using VSEARCH (version 2.13.4) [19], resulting in the production of high-quality clean tags. These high-quality sequences were clustered into amplicon sequence variants (ASVs) using UPARSE (v7.1) for bioinformatics analysis, employing a 97% sequence similarity threshold. Taxonomic classifications were assigned by selecting representative sequences from the ASVs and screening each ASV against the Ribosomal Database Project (RDP) database [20].

Gut microbiota and muscle transcriptome data were processed via the Personal Ge-nomics Cloud Platform, available at https://www.genescloud.cn/ (accessed on 30 April 2025), except the interaction network. A percent stacked column chart was constructed to visualize the species composition at the phylum or genus level of fish residing in different environments. Alpha diversity indices, including Good’s coverage, Pielou’s evenness, Shannon diversity, and Simpson diversity, were calculated using QIIME2 (version 2022.11) to assess the richness and diversity of gut microbial communities in each sample. Principal coordinate analysis (PCoA) and the unweighted pair-group method with arithmetic means (UPGMA) were used to illustrate shifts in microbial community structure among samples, whereas linear discriminant analysis effect size (LEfSe) identified microbial taxa with differing abundance between groups.

ASVs with a cumulative relative abundance < 0.01% were excluded from the network analysis. Interspecies interaction networks for each group were constructed using Spearman correlation analysis on the platform https://www.omicstudio.cn/tool (accessed on 11 May 2025), with thresholds set at an absolute correlation coefficient ≥ 0.85 and a *p*-value < 0.05. Network visualizations were drawn using Gephi (version 0.9.7). To explore the associations between gut microbiota, fatty acid metabolism, and immune status, ASVs were organized into modules using the weighted correlation network analysis (WGCNA) package (version 1.72-5) in R (version 4.4.2). A heatmap, based on Spearman’s rank correlations and generated using the heatmap package (version 1.0.12), was employed to elucidate the relationships between modules and factors associated with fatty acid metabolism and immunity. The species compositions of modules were depicted via the plot_pie in the ggplot2 package (version 3.5.1).

### 2.6. Statistical Analysis

The data were analyzed using SPSS (version 23.0, Chicago, IL, USA), presenting results as means ± standard error (SEM). An independent-sample T-test determined differences in muscle proximate composition, amino acid profile, fatty acid content, texture, and parameters of α-diversity of gut microbiota between cultured and wild *C. nasus*, with significance at *p* < 0.05 and extreme significance at *p* < 0.01. Gut microbiota and muscle transcriptome data were processed via the Personal Genomics Cloud Platform, available at https://www.genescloud.cn/ (accessed on 11 May 2025), except the interaction network. The interaction network of gut microbiota at the genus level was constructed at https://www.omicstudio.cn/tool (accessed on 11 May 2025) and was drawn using Gephi (version 0.9.7).

## 3. Results

### 3.1. Muscle Physical and Chemical Properties

#### 3.1.1. Muscle Proximate Composition and Meat Quality

The muscle lipid content of wild *C. nasus* was obviously higher than that of the cultured population (Table 1) (*p* < 0.05), whereas the muscle moisture and ash content were significantly lower (*p* < 0.05). There was no significant difference in the muscle crude protein content between the two populations (*p* > 0.05). The shear force, drip loss, and cooking loss of muscle from wild *C. nasus* were significantly lower compared to those from cultured fish (*p* < 0.05). Conversely, no significant differences were found in other parameters related to meat quality between the wild and cultured groups (*p* > 0.05).

#### 3.1.2. Muscle Amino Acid Composition

In the study of muscle tissue from wild and cultured *C. nasus*, no obvious differences were found in the nine essential amino acids (EAAs) (*p* > 0.05). However, serine and proline concentrations were obviously higher in the cultured fish among eight non-essential amino acids (NEAAs) (*p* < 0.05), whereas the other NEAAs showed no significant differences (*p* > 0.05). Furthermore, the total EAA (∑EAA), total NEAA (∑NEAA), and total AA (∑TAA) contents were not found to significantly differ between the groups (*p* > 0.05) (Table 2).

#### 3.1.3. Muscle Fatty Acid Composition

Regarding the saturated fatty acids (SFAs) in muscle tissue, as presented in Table 3, the levels of C14:0, C15:0, C17:0, and C20:0 were significantly higher in the cultured group than in the wild group (*p* < 0.05), whereas the opposite trend was observed for C16:0 (*p* < 0.05). The monounsaturated fatty acid C18:1n9c was the most abundant in the muscle tissue of both groups, with significantly higher levels in the wild group compared to the cultured group (*p* < 0.05). A similar trend was observed for C22:1n9, while the levels of C18:1n9t and C20:1 were obviously higher in the cultured fish (*p* < 0.05). In the muscle tissue of both wild and cultured *C. nasus*, the major polyunsaturated fatty acids (PUFAs) were identified as C22:6n3 (DHA) and C18:2n6c (LA), respectively, with statistically significant differences observed between the two groups (*p* < 0.05). The concentrations of C18:3n3 (ALA), C20:2, and C20:4n6 (ARA) were significantly elevated in the cultured population (*p* < 0.05). Conversely, the wild fish exhibited an obviously higher concentration of C22:1n9 in muscle tissue (*p* < 0.05). Notably, C20:3n6 (DHLA) was exclusively detected in the cultured population. The total omega-6 polyunsaturated fatty acid (∑n-6 PUFA) content of the wild group was obviously lower than that of the cultured group (*p* < 0.05), while no obvious difference was found in the total omega-3 PUFA (∑n-3 PUFA) content between the two groups (*p* > 0.05). Moreover, ∑n-3 PUFA/n-6 PUFA in the cultured group was significantly lower than that in the wild group (*p* < 0.05), with the specific value in the wild group being approximately six times greater than that in the cultured group.

#### 3.1.4. Muscle Antioxidant Capacity

**Figure 2:**
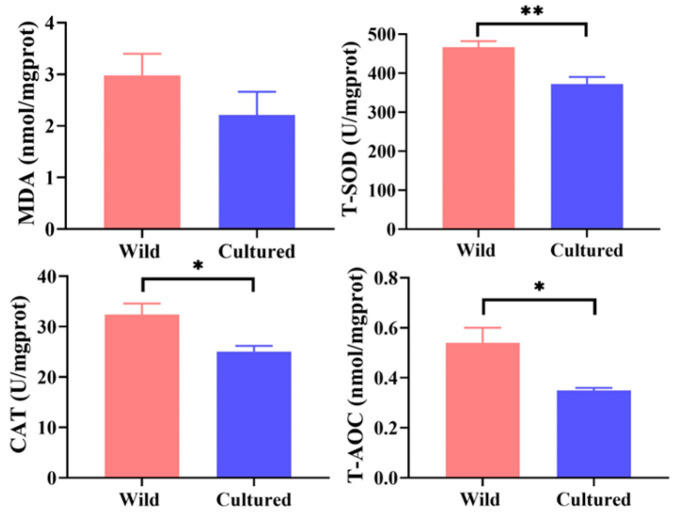
Antioxidant capacity of cultured and wild *C. nasus*. MDA, malondialdehyde; T-SOD, total superoxide dismutase; CAT, catalase; T-AOC, total antioxidant capacity. * represents significant difference (*p* < 0.05); ** represents extremely significant difference (*p* < 0.01).

Compared with the cultured group, the wild *C. nasus* exhibited significantly higher activities of T-SOD, CAT, and T-AOC in the muscle (*p* < 0.05). There was no significant difference in the muscle MDA content between the two groups (*p* > 0.05) (Figure 2).

**Figure 3:**
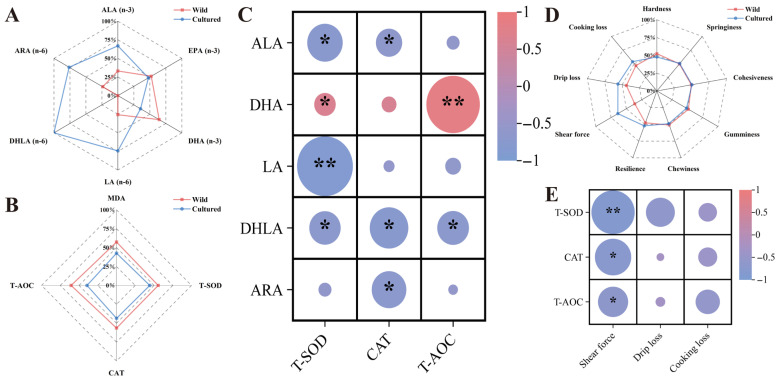
The correlation between n-3/n-6 PUFAs, antioxidant capacity, and meat quality indicators. (**A**,**B**,**D**) The radar chart representing the differences in composition of n-3 and n-6 PUFAs, antioxidant status, and meat quality in the muscle between wild and cultured *C. nasus*. (**C**,**E**) The heatmap showing the correlations between antioxidant capacity, n-3 and n-6 PUFAs, and meat quality. * represents significant difference (*p* < 0.05); ** represents extremely significant difference (*p* < 0.01).

As illustrated in Figure 3A,B,D, the radar plot effectively depicts the status of muscle n-3 and n-6 PUFAs, antioxidant capacity, and meat texture between the two groups. The muscle n-6 PUFAs, including LA, DHLA, and ARA, as well as the n-3 PUFA ALA, demonstrated a negative correlation with antioxidant enzymes. However, DHA content was positively correlated with T-SOD and T-AOC activities (*p* < 0.05) (Figure 3C). In addition, the activities of antioxidant enzymes were negatively correlated with shear force (*p* < 0.05) (Figure 3E).

### 3.2. Muscle Transcriptomic Analysis

Cluster analysis (Figure S1A) indicated that all expressed genes were organized into nine distinct clusters. Within two of these clusters, gene expression levels in the cultured *C. nasus* exceeded those observed in their wild counterpart, whereas the inverse was observed in the remaining seven clusters. Principal component analysis (PCA) (Figure S1B) demonstrated that while the gene expression patterns of the two groups were largely similar, notable differences were present. The volcano plot (Figure S1C) identified a total of 1806 differentially expressed genes (DEGs). Of these, 690 genes (38.21%) showed higher expression levels in the cultured *C. nasus* than the wild, whereas 1116 genes (61.79%) exhibited lower expression levels in the cultured population relative to the wild.

**Figure 4:**
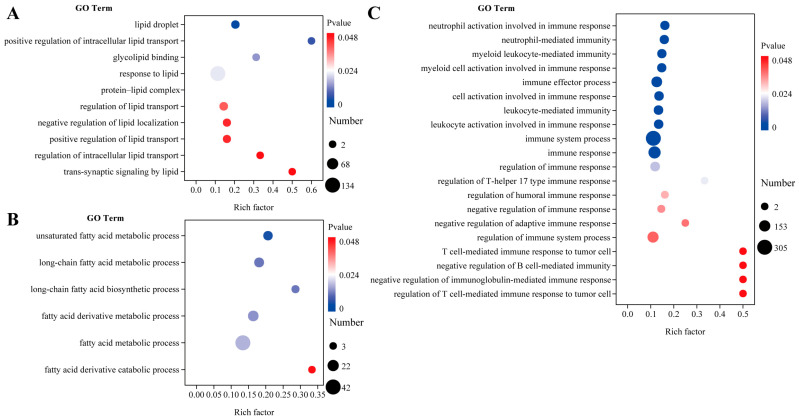
Lipid and fatty acid metabolism and immune response in the muscles of cultured and wild *C. nasus*. (**A**) Differential GO terms related to lipid metabolism. (**B**) Differential GO terms related to fatty acid metabolism. (**C**) Differential GO terms related to immune response. Wild was set as the control group. Cultured was set as the treatment group.

To enhance our understanding of the differences in fatty acid metabolism and immune function in the muscle tissue of wild versus cultured *C. nasus*, we performed a GO enrichment analysis focusing on lipid and fatty acid metabolism as well as immune response (Figure 4). In comparison to the wild *C. nasus*, the farmed fish exhibited an enrichment of GO terms related to these processes, with counts of 10 (Figure 4A), 6 (Figure 4B), and 20 (Figure 4C), respectively. Notably, the terms with the highest enrichment factors and a *p* < 0.05 value included positive regulation of intracellular lipid transport, fatty acid derivative catabolic process, T cell-mediated immune response to tumor cells, negative regulation of B cell-mediated immunity, negative regulation of immunoglobulin-mediated immune response, and regulation of T cell-mediated immune response to tumor cells.

**Figure 5:**
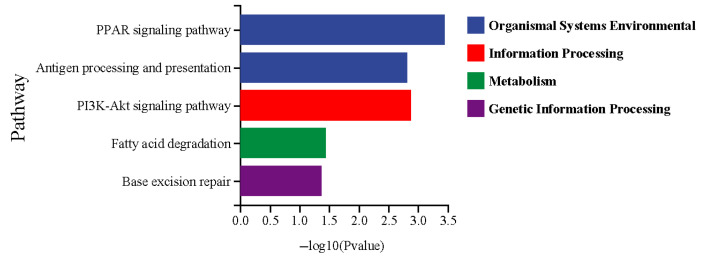
The KEGG pathways related to lipid and fatty acid metabolism and immune response. Wild was set as the control group. Cultured was set as the treatment group.

**Figure 6:**
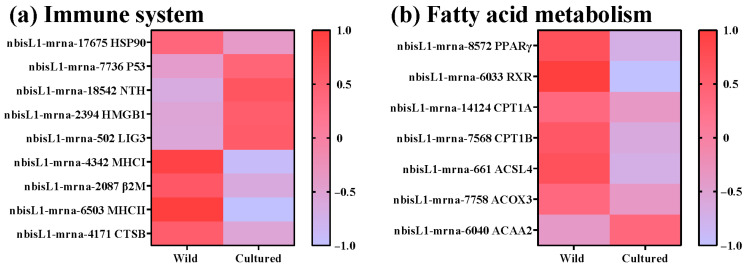
DEGs in the KEGG pathways regulating the immune system and fatty acid metabolism. (**a**) Genes related to the immune system: HSP90, Heat Shock Protein 90; P53, Tumor Protein P53; NTH, Endonuclease III-like DNA glycosylase; HMGB1, High Mobility Group Box 1; LIG3, DNA Ligase III; MHC, Major Histocompatibility Complex Class; β2M, Beta-2 Microglobulin; CTSB, Cathepsin B. (**b**) Genes related to fatty acid metabolism: PPARγ, Peroxisome proliferator-activated receptor gamma; RXR, Retinoid X receptor; CPT1, Carnitine palmitoyltransferase 1; ACSL4, Acyl-CoA synthetase long-chain family member 4; ACOX3, Acyl-CoA oxidase 3; ACAA2, Acetyl-CoA acyltransferase 2.

To further elucidate the principal signaling pathways implicated in fatty acid metabolism and the immune system, a KEGG enrichment analysis was conducted (Figure 5). The DEGs were enriched in five KEGG pathways, such as the PPAR signaling pathway, antigen processing and presentation, the PI3K-Akt signaling pathway, fatty acid degradation, and base excision repair. To identify specific genes related to fatty acid metabolism and the immune system, a combined GO and KEGG enrichment analysis was performed to select DEGs common to both analyses (Figure 6). Nine DEGs related to the immune system were observed, including HSP90, P53, NTH, HMGB1, LIG3, MHC I, β2M, MHC II, and CTSB (Figure 6a). Among these, HSP90, MHC I, β2M, MHC II, and CTSB were upregulated in the wild *C. nasus*, whereas P53, NTH, HMGB1, and LIG3 were upregulated in the cultured. Additionally, seven DEGs involved in fatty acid metabolism were identified, including PPARγ, RXR, CPT1A, CPT1B, ACSL4, ACOX3, and ACAA2 (Figure 6b). Compared with the wild group, ACAA2 was upregulated in the cultured group, while PPARγ, RXR, CPT1A, CPT1B, ACSL4, and ACOX3 were downregulated.

### 3.3. Gut Microbiota Analysis

At the phylum level, the gut microbiota of wild *C. nasus* was predominantly composed of Proteobacteria, Bacteroidota, and Actinobacteriota, in descending order of abundance. Conversely, in cultured *C. nasus*, the gut microbiota of was primarily composed of Firmicutes_A, Proteobacteria, and Actinobacteriota, also in descending order (Figure S2A). Among the top 15 phyla by abundance, Firmicutes_A and Actinobacteriota were elevated in the cultured *C. nasus* compared to the wild, whereas Proteobacteria and Bacteroidota were obviously diminished in the cultured group (*p* < 0.05) (Figure S2C).

At the genus level, the gut microbiota of wild *C. nasus* mainly comprised *Pseudomonas_E*, *Aquabacterium_B*, and *Stenotrophomonas_A*, in descending order. In contrast, the gut microbiota of cultured *C. nasus* was predominantly composed of *Clostridium_T*, *Methylobacterium*, and *Pseudomonas_E*, in descending order (Figure S2B). Among the top 15 genera by abundance, only *Clostridium_T* exhibited a significantly higher abundance in the cultured population than the wild (*p* < 0.05), whereas the abundances of *Aquabacterium_B*, *Stenotrophomonas_A*, *Herbaspirillum*, *Sphingomonas_L*, *Comamonas_F*, *Variovorax*, *Sphingobacterium*, *Clostridium_T*, and *Pseudomonas_E* were obviously lower in the cultured fish compared to the wild group (*p* < 0.05) (Figure S2D).

The Pielou, Shannon, and Simpson indices were elevated in wild *C. nasus* compared to the cultured (Figure S3A) (*p* < 0.05). Conversely, Good’s coverage was lower in wild *C. nasus* relative to cultured, whereas the other three indices exhibited opposite trends (*p* < 0.05). Furthermore, the PCoA plot (Figure S3B) demonstrated a distinct separation in gut microbiota between wild and cultured *C. nasus.*

**Figure 7:**
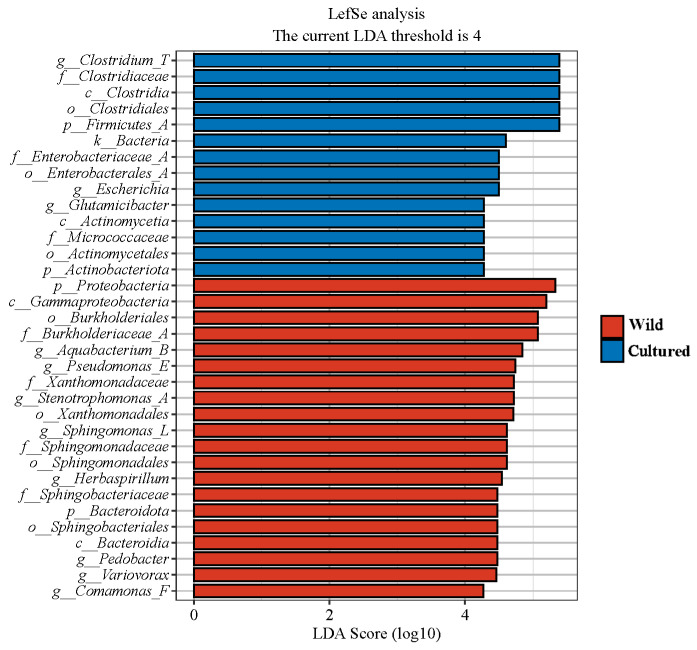
Linear discriminant analysis effect size (LEfSe) analysis of gut microbiota composition of *C. nasus* between cultured and wild environments (LDA > 4).

A LEfSe analysis was performed on the microbiota of wild and cultured *C. nasus* of the Yangtze River, with the LDA score threshold set at 4. As illustrated in Figure 7, the gut microbiota of cultured *C. nasus* revealed the presence of two phyla (Firmicutes_A and Actinobacteriota), two classes (Clostridia and Actinomycetia), three orders (Clostridiales, Enterobacterales_A, and Actinomycetales), three families (Clostridiaceae, Enterobacteriaceae_A, and Micrococcaceae), and three genera (*Clostridium_T*, *Escherichia*, and *Glutamicibacter*) as biomarkers. In contrast, the gut microbiota of wild *C. nasus* was characterized by two phyla (Proteobacteria and Bacteroidota), two classes (Gammaproteobacteria and Bacteroidia), four orders (Burkholderiales, Xanthomonadales, Sphingomonadales, and Sphingobacteriales), four families (Burkholderiaceae_A, Xanthomonadaceae, Sphingomonadaceae, and Sphingobacteriaceae), and eight genera (*Aquabacterium_B*, *Pseudomonas_E*, *Stenotrophomonas_A*, *Sphingomonas_L*, *Herbaspirillum*, *Pedobacter*, *Variovorax*, and *Comamonas_F*) identified as biomarkers.

**Figure 8:**
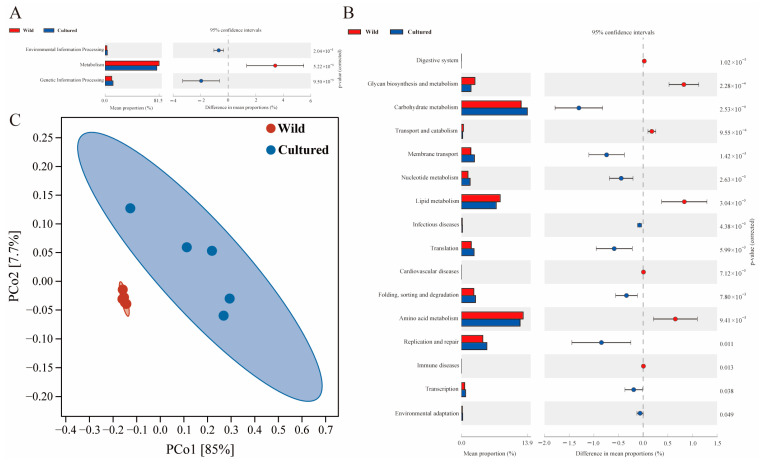
Metabolic functional profiles of *C. nasus* gut microbiota between cultured and wild environments. (**A**,**B**) KEGG level 1 and level 2 metabolic functional analysis of the gut microbiota in *C. nasus* from cultured and wild environments; (**C**) PCoA plot for the differences in functional units of the gut microbiota between cultured and wild *C. nasus*.

At KEGG level 1, the gut microbiota of *C. nasus* was primarily associated with essential functional categories, including metabolism, genetic information processing, and environmental information processing (Figure 8A). Moreover, at KEGG level 2, notable differences were identified in the distribution of diet-related functional categories between cultured and wild *C. nasus*, specifically in carbohydrate metabolism, amino acid metabolism, and lipid metabolism (*p* < 0.05) (Figure 8B). The category pertaining to replication and repair, which was related to the immune system, was also identified. Additionally, the PCoA plot distinctly demonstrated that the gut microbiota in wild and cultured *C. nasus* were clearly distinguishable (Figure 8C).

### 3.4. Correlation Analysis

**Figure 9:**
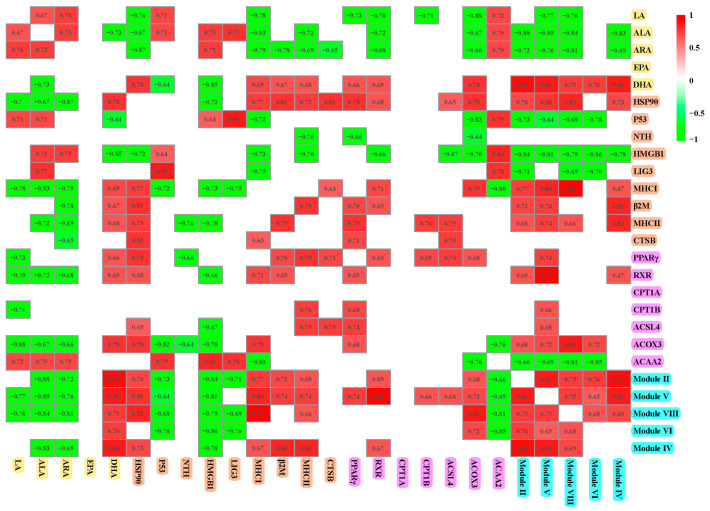
Spearman’s correlations between modules and factors associated with fatty acid metabolism and immunity. Only significant correlations (*p* < 0.05) are shown.

As illustrated in Figure 9, a positive correlation was observed among LA, ALA, and ARA, whereas DHA exhibited a negative correlation with ALA. LA, ALA, and ARA demonstrated negative correlations with HSP90, MHC I, RXR, and ACOX3 but positively related to ACAA2. ARA negatively related to β2M and CTSB. DHA showed negative correlations with P53 and HMGB1, yet it was positively correlated with indices related to immune defense, such as HSP90, MHC I, β2M, and MHC II, as well as fatty acid metabolism markers including PPARγ, RXR, and ACOX3. HSP90 was positively correlated with MHC I, β2M, MHC II, CTSB, PPARγ, RXR, ACSL4, and ACOX3, while exhibiting a negative correlation with HMGB1. P53 was negatively related to HMGB1, LIG3, and ACAA2, and positively related to MHC I and ACOX3. PPARγ negatively correlated with NTH and positively related to β2M, MHC II, CTSB, RXR, CPT1B, ACSL4, and ACOX3. Modules II, V, and VIII were positively correlated with each of the other four modules. These modules also exhibited negative correlations with ALA, ARA, P53, HMGB1, and ACAA2 while showing positive correlations with DHA, HSP90, MHC I, MHC II, and ACOX3. Additionally, modules VI and IV were positively related to DHA and negatively related to HMGB1.

**Figure 10:**
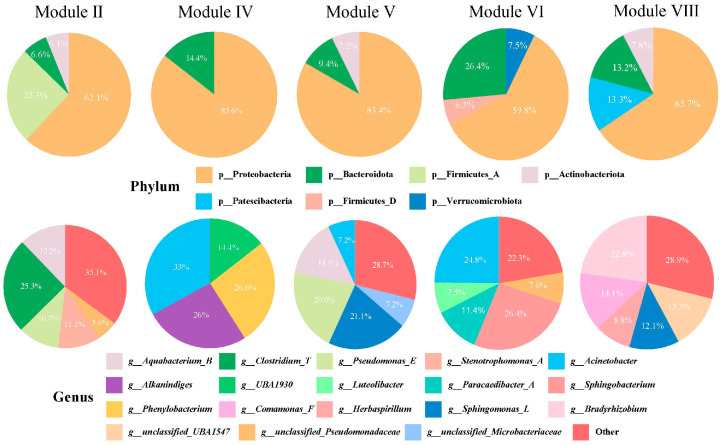
Composition of the dominant modules as identified by WGCNA at the phylum and genus levels.

To investigate species composition within each module, pie charts were used to illustrate the proportion representation in the module (Figure 10). At the phylum level, all five modules included Proteobacteria and Bacteroidota, with Proteobacteria constituting the predominant proportion across all modules. At the genus level, microorganisms that comprised more than 20% of each module included *Clostridium_T* (module II), *Sphingomonas_L* and *Pseudomonas_E* (module V), *Bradyrhizobium* (module VIII), *Sphingobacterium* and *Acinetobacter* (module VI), and *Acinetobacter*, *Phenylobacterium* and *Alkanindiges* (module IV). Notably, Proteobacteria, Bacteroidota, *Sphingomonas_L*, and *Pseudomonas_E* served as biomarkers in the wild *C. nasus*, whereas *Clostridium_T* was identified as the biomarker in the cultured group.

**Figure 11:**
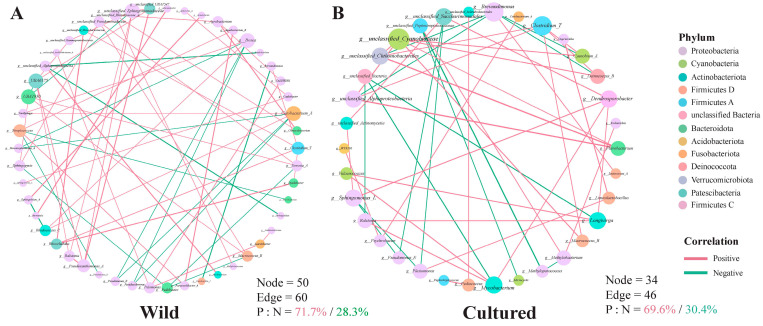
Gut microbiota genus-level interaction network. (**A**) wild group; (**B**) cultured group. Each node represents a genus. Node colors denote genera belonging to various major phyla. A green edge represents a negative interaction between two nodes, while a red edge represents a positive interaction.

To explore the relationship between the gut bacterial communities in the wild and cultured *C. nasus*, microbial networks were constructed (Figure 11). The gut microbial network for the cultured fish possessed 34 nodes and 46 edges (Figure 11B), while the network for the wild comprised 50 nodes and 60 edges (Figure 11A). The rate of positive correlations within the network of the cultured population was decreased compared to the wild. Integrating findings from modular analysis, it was observed that in the gut of wild *C. nasus*, *Sphingomonas_L* exhibited a positive correlation with *Caulobacter*, while *Pseudomonas_E* showed negative correlations with *Bosea* and *Cetobacterium_A*. Additionally, *Bradyrhizobium* was negatively related to *unclassified_Alphaproteobacteria*, and *Acinetobacter* was positively related to *Sphingobium_A*. In the gut of cultured *C. nasus*, *Clostridium_T* demonstrated positive correlations with the abundance of *Cyanobium_A*, *Deinococcus_B*, *Macrococcus_B*, *unclassified_Alphaproteobacteria*, and *unclassified_Chthoniobacterales*. Furthermore, *Sphingomonas_L* exhibited negative correlations with *Plesiomonas* and *Pseudomonas_E* and positive correlations with *unclassified_Actinomycetia* and *Dendrosporobacter*. Moreover, *Pseudomonas_E* was also negatively correlated with *unclassified_Saccharimonadales*.

## 4. Discussion

The body composition of wild and cultured fish is widely recognized to differ due to environmental and dietary factors. In this study, cultured *C. nasus* demonstrated higher moisture and ash contents and lower lipid content in muscle compared to their wild counterparts, aligning with findings reported by Tang et al. [21,22]. The muscle crude protein content in both wild and cultured *C. nasus* was similar, a trend also found in the studies of bighead carp (*Hypophthalmichthys nobilis*) [16] and Yellow River carp (*Cyprinus carpio* haematopterus) [23]. In the cultured population, *C. nasus* showed a notable decrease in muscle lipid content, unlike other farmed species, such as turbot (*Oncorhynchus mykiss*), bream (*Sparus aurata*), and crayfish (*Procambarus clarkii*), which exhibit higher lipid levels than their wild counterparts but lower n-3 LC-PUFA [24,25]. Furthermore, wild fish have a better n-3/n-6 fatty acid ratio compared to farmed fish [16,26]. Additionally, the regulation of osmotic pressure resulted in a higher absorption rate of calcium ions in cultured *C. nasus* in freshwater environments compared to wild *C. nasus*, which had recently migrated from the sea to the Taizhou section of the Yangtze River. This increased calcium absorption led to enhanced bone density and a corresponding rise in ash content [21]. Meat texture was significantly influenced by its nutritional composition [27]. In the current study, it was observed that the shear force in the muscle of cultured *C. nasus* was elevated, aligning with findings related to high ash content. An increase in muscle drip loss would lead to greater liquid outflow and the subsequent loss of soluble nutrients, thereby diminishing the quality and flavor of the meat [28]. Notably, the muscle drip loss and cooking loss in cultured *C. nasus* were lower than those observed in wild *C. nasus*, potentially contributing to the higher muscle shear force in the cultured group.

Muscle amino acid and fatty acid profiles also served as crucial indicators for evaluating muscle quality. In the present study, the levels of muscle serine and proline in cultured *C. nasus* were higher compared to their wild counterparts, corroborating the findings of Tang et al. [21]. Proline accumulation was involved in osmoregulation [29,30], which may explain the increased muscle ash content in cultured *C. nasus*. Furthermore, variations in serine content may affect fatty acid metabolism [31]. This partially accounts for the observed differences in fatty acid composition within the muscle tissues of the two groups. The degree of fatty acid saturation could influence the fat hardness, thereby affecting meat quality [32]. In this study, SFA content in muscle between cultured and wild *C. nasus* was similar, potentially explaining the lack of significant variation in muscle hardness. Among the SFAs, C16:0 was identified as the predominant fatty acid, while C18:1n9c emerged as the leading monounsaturated fatty acid (MUFA) across all *C. nasus* muscle samples. These findings align with previous research on trout (*Oncorhynchus mykiss*) [26] and bighead carp [16]. Although certain SFAs, like C14:0 and C16:0, have been associated with an increased risk of cardiovascular disease, their impact—whether be beneficial, neutral, or harmful—depends on the dietary level of n-3 PUFA [33,34]. In this study, while no significant difference was observed in ∑ n-3 PUFA content in muscle between the two groups, the ratio of ∑ n-3 PUFA to ∑ n-6 PUFA was higher in wild *C. nasus* compared to their cultured counterparts. Notably, within the n-3 PUFAs, EPA and DHA are critical indicators for assessing the nutritional value of fatty acids, as they play significant roles in nutrition supply, disease prevention, and health promotion [35,36]. Remarkably, the muscle tissue of wild *C. nasus* exhibited a higher DHA content than that of cultured *C. nasus*. Similar findings have been reported in studies on Rio Grande silvery minnows (*Hybognathus amarus*) [37] and bighead carp [16]. From this perspective, wild *C. nasus* possess a relatively higher nutritional value, likely attributable to the more comprehensive and balanced fatty acid composition of their natural diet. On the other hand, previous studies have indicated that many eicosanoids derived from n-6 PUFAs, including prostaglandins, thromboxanes, leukotrienes, and their respective metabolites, could transmit inflammatory signals and contribute to the onset and progression of inflammation [38,39]. Based on the fatty acid profile observed in muscle tissue, we hypothesized that the immune status of wild fish may be more robust than that of cultured fish.

This study investigated the differences in immune status between the muscles of wild and cultured *C. nasus*. Our findings revealed that antioxidant indicators of wild *C. nasus* were elevated compared to those in cultured *C. nasus* in the muscles, corroborating the results reported by Tang et al. [22]. The antioxidant capacity of fish appeared to be influenced by their environmental conditions. Typically, carnivorous fish residing in the benthic layer exhibit greater antioxidant capacity than omnivorous or herbivorous fish inhabiting the pelagic or epipelagic zones [40]. Wild *C. nasus*, which inhabited deeper waters with abundant food resources, demonstrated this through their muscle fatty acid composition. The antioxidant capacity of cultured *C. nasus* may be inferior to that of their wild counterparts, perhaps due to differences in the nutritional composition of their daily feed, particularly in fatty acids. Correlation analysis suggested that muscle n-3/n-6 PUFAs may play a critical role in modulating antioxidant capacity. Variations in antioxidant status among animals or muscle tissues can influence proteolysis, subsequently affecting meat quality attributes such as tenderness and water-holding capacity [41,42]. In this study, wild *C. nasus* exhibited lower shear force and higher antioxidant capacity compared to cultured *C. nasus*, with correlation analysis revealing a significant negative relationship between these two parameters.

To gain further insights into the molecular mechanisms underlying fatty acid metabolism and immune response in *C. nasus* muscle, transcriptome analysis was conducted. Muscle tissue has traditionally been regarded as a significant metabolic organ rather than an immune organ. However, this study found that immune-related biological processes were more prominently enriched, indicating that muscles may be important in immune defense. Analysis of genes in these three biological processes identified five relevant KEGG signaling pathways, three of which were related to the immune system. Moreover, in the muscle tissues of two *C. nasus* groups, different gene expression patterns related to the immune system were observed. This suggested potential differences in immune response and stress adaptation capabilities between fish from different environments. It was hypothesized that external factors, such as varying environmental pressures and pathogen exposure encountered by wild and cultured organisms, as well as internal factors, including fatty acid composition, may contribute to these observed differences. Certain transcription factors that are indicative of DNA damage, such as NTH [43], HMGB1 [44], and LIG3 [45], serve as pivotal regulators in promoting the expression of downstream pro-inflammatory cytokines. These genes exhibited significant upregulation in the muscle tissue of cultured *C. nasus*. The MHC I and II pathways facilitate the presentation of antigen fragments to the immune system through a series of biochemical reactions [46]. Key genes within these pathways, such as MHC I, β2M, MHC II and CTSB, were markedly upregulated in the muscles of wild *C. nasus*. Transcriptome analysis of muscle tissue revealed that the immune status of wild *C. nasus* was more robust than that of cultured *C. nasus*, aligning with findings from antioxidant enzyme activity assays.

PPARγ, a well-researched subtype of the PPAR family, is vital for adipocyte differentiation and fatty acid and glucose metabolism [47,48]. Upon binding to its ligand and forming a heterodimer with the retinoic acid X receptor (RXR), PPARγ is considered to regulate gene transcription associated with metabolism and inflammation [49]. The activation of PPARγ by PUFAs, particularly EPA and DHA, has been extensively described in earlier studies [50,51,52]. For example, DHA has been evaluated for its ability to mitigate the harmful effects of LA on retinal pigment epithelial cells [52]. Pretreatment with 50–100 µM DHA was found to inhibit LA-induced production of monocyte chemoattractant protein 1 (MCP-1) and to downregulate NF-κB activation in a dose-dependent manner [52]. A diet with a low n-6/n-3 PUFA ratio was more effective in reducing inflammation in murine models of colitis via PPARγ activation [53]. Notably, this study observed an upregulation of PPARγ and RXR in the wild group, which correlated with high DHA content and a low n-6/n-3 PUFA ratio in their muscle tissue. Correlation analysis further revealed that PPARγ expression was negatively related to LA and positively related to DHA. Additionally, PPARγ activation was shown to upregulate the expression of CPT1 and promote fatty acid oxidation [54]. Among the genes involved in fatty acid oxidation, acyl-CoA synthetase long-chain family member 4 (ACSL4) plays a crucial role by converting free fatty acids into fatty acyl-CoAs, which subsequently enter the oxidation process [55]. Acyl-CoA oxidases, specifically ACOX1, ACOX2, and ACOX3, facilitate the oxidation of fatty acyl-CoA by transferring two hydrogens atoms to flavin adenine dinucleotide (FAD), resulting in the formation of enoyl-CoA and FADH2 [56]. In the muscle tissue of wild *C. nasus*, these genes involved in fatty acid β-oxidation were markedly upregulated. This upregulation suggested not only that wild *C. nasus* exhibits a higher energy demand than its cultured counterparts, but it may also be linked to the development of the muscle fatty acid profile, as inferred from correlation analyses.

There is a growing body of evidence recognizing the gut microbiota’s substantial influence on immune and metabolic functions throughout the body, extending beyond the gut [57]. Therefore, it is imperative to explore the microbiota composition and its association with fatty acid metabolism and immune status in the muscle. The host environment is a critical determinant in shaping the gut microbiome in fish [58]. In the present study, gut microbiota structure between wild and cultured *C. nasus* was obviously different, as evidenced by alpha and beta diversity analyses. Proteobacteria were identified as the dominant microbial group, corroborating findings from previous research on *C. nasus* [59,60]. Firmicutes_A and the genus *Clostridium_T* in the cultured group and the phylum Proteobacteria and genus *Pseudomonas_E* in the wild group were identified as biomarkers, which suggested differences in the environmental conditions of the two groups. The differing microbial profiles between wild and cultured *C. nasus* were likely due to intensive aquaculture practices. Antibiotics may suppress beneficial bacteria, while immunostimulants and probiotics might selectively promote specific taxa like *Clostridium_T*. These practices improve disease resistance but may reduce microbial diversity, as evidenced by lower α-diversity in cultured fish. This dysbiosis carries dual effects: better pathogen control but potential negative impacts on metabolism, particularly lipid assimilation and immune homeostasis. This study found that dominant genus differences between cultured and wild *C. nasus* affect their metabolic and immune status, with wild fish showing increased pathways for lipid metabolism and immune diseases, suggesting the gut microbiota’s role in influencing fatty acid metabolism and immune function in muscle tissues.

To elucidate the impact of gut microbial co-occurrence patterns on muscle fatty acid metabolism and immune processes, a modular analysis was employed. In this study, the five selected modules demonstrated positive correlations, indicating that they likely perform similar functions. This hypothesis was further supported by correlation analyses between the modules and various indicators related to fatty acid metabolism and immunity. *Clostridium_T* was the most dominant species in module II. Certain *Clostridium* spp., like *Clostridium butyricum*, are known to provide nutritional benefits to the host, particularly in terms of fatty acids and vitamins [61]. *Pseudomonas_E* and *Sphingomonas_L* accounted for the largest proportions and were also identified as dominant genera in the gut of wild *C. nasus*. Notably, *Pseudomonas* aeruginosa has been reported to synthesize unsaturated fatty acids [62]. In *P. aeruginosa*, the level of unsaturated fatty acids (UFAs) was higher than saturated fatty acids (SFAs), with a UFA-to-SFA ratio of 1.8 [63]. Therefore, *Pseudomonas_E* is highly likely to participate in the metabolism of unsaturated fatty acids. Glycosphingolipids (GSLs) derived from *Sphingomonas paucimobilis* have been shown to activate NKT cells and enhance the host’s immune response [64]. *Bradyrhizobium* emerged as the predominant genus within module VIII. Prior research has shown that lipopolysaccharides (LPSs) isolated from *Bradyrhizobium* strains exhibit a strong inhibitory effect on MD-2/TLR4 activation by toxic enteric bacterial LPS, indicating that the bacterium’s potential for immune protection [65]. An analysis of the gut microecological network at the genus level was conducted to explore the interactions among the gut microbiota in each group of *C. nasus*. The number of links (edges) and the rate of negative interactions serve as indicators of the complexity [66]. A decline in complexity increases the susceptibility to invasion by external strains [67]. In this study, the limited number of links within the gut microbiota of cultured *C. nasus* may heighten the fish’s vulnerability to disease threats. Moreover, in the gut of wild *C. nasus*, no correlation was observed between *Sphingomonas_L* and *Pseudomonas_E*, while in the gut of cultured *C. nasus*, these two microbial genera exhibited a competitive relationship. Environmental changes that led to a depletion of resources, such as nutrients in the gut, prompted microorganisms to compete with genera with which they were not previously in competition to secure limited resources [68]. This finding suggested a potential deficiency in the nutritional composition of the feed used for artificially cultured *C. nasus* and underscores the necessity for further research into the nutritional requirements of *C. nasus*.

## 5. Conclusions

This study elucidates differences in fatty acid metabolism and immune status between cultured and wild *C. nasus*. Wild *C. nasus* exhibited a higher DHA level and a better n-3/n-6 PUFA ratio, indicating superior nutritional value. Furthermore, wild *C. nasus* had a stronger immune status. A positive correlation was demonstrated between DHA, n-3/n-6 PUFA ratio, and immune status in *C. nasus* muscle. The gut microbiota analysis revealed *Pseudomonas_E* and *Clostridium_T* as the predominant microbial genera in wild and cultured *C. nasus*, respectively, both of which contributed to the regulation of fatty acid metabolism and immune processes, as indicated by the modular analysis. These findings contribute to a deeper understanding of the interplay between fatty acid metabolism, immune status, and gut microbiota in *C. nasus* and underscore the potential for developing enhanced artificial feed formulations.

## Figures and Tables

**Table 1:** Muscle proximate composition and meat quality of cultured and wild *C. nasus*.

Item	Cultured	Wild	*p* Value
Proximate composition (Wet weight)			
Moisture	64.13 ± 1.38	51.41 ± 1.83	*
Crude protein	20.05 ± 0.49	21.95 ± 1.26	ns
Lipid	4.14 ± 0.55	10.85 ± 0.80	*
Ash	1.67 ± 0.06	1.38 ± 0.07	*
Texture			
Hardness	2401.06 ± 325.48	2637.10 ± 256.52	ns
Springiness	0.38 ± 0.02	0.38 ± 0.01	ns
Cohesiveness	0.39 ± 0.02	0.38 ± 0.02	ns
Gumminess	934.80 ± 147.20	994.42 ± 73.71	ns
Chewiness	361.81 ± 62.49	376.90 ± 26.89	ns
Resilience	0.23 ± 0.02	0.21 ± 0.01	ns
Shear force	1.05 ± 0.10	0.59 ± 0.03	*
Water-holding capacity			
Drip loss	13.66 ± 1.10	10.71 ± 0.37	*
Cooking loss	20.78 ± 1.16	17.92 ± 0.33	*

The data are shown as mean ± SEM; * represents significant difference (*p* < 0.05); ns: non-significant (*p* > 0.05).

**Table 2:** Muscle amino acid composition of cultured and wild *C. nasus* (g/100 g).

Amino Acid	Cultured	Wild	*p* Value
Essential amino acids			
Histidine	0.323 ± 0.008	0.311 ± 0.006	ns
Threonine	0.729 ± 0.015	0.701 ± 0.005	ns
Arginine	1.086 ± 0.024	1.151 ± 0.005	ns
Valine	0.883 ± 0.020	0.874 ± 0.007	ns
Methionine	0.578 ± 0.024	0.561 ± 0.008	ns
Phenylalanine	0.806 ± 0.025	0.785 ± 0.014	ns
Isoleucine	0.782 ± 0.020	0.771 ± 0.013	ns
Leucine	1.466 ± 0.043	1.437 ± 0.017	ns
Lysine	1.772 ± 0.060	1.711 ± 0.029	ns
Non-essential amino acids			
Aspartic acid	2.104 ± 0.053	2.008 ± 0.027	ns
Glutamic acid	3.121 ± 0.078	3.045 ± 0.036	ns
Serine	0.739 ± 0.018	0.688 ± 0.005	*
Glycine	1.004 ± 0.024	0.919 ± 0.029	ns
Alanine	1.149 ± 0.019	1.118 ± 0.003	ns
Tyrosine	0.467 ± 0.015	0.453 ± 0.004	ns
Cystines	0.023 ± 0.001	0.022 ± 0.001	ns
Proline	0.399 ± 0.035	0.291 ± 0.011	*
∑EAA	8.424 ± 0.235	8.031 ± 0.087	ns
∑NEAA	9.007 ± 0.199	8.543 ± 0.055	ns
∑TAA	17.431 ± 0.429	16.844 ± 0.134	ns

The data are shown as mean ± SEM. EAA, essential amino acid; NEAA, non-essential amino acid, TAA, total amino acid. * represents significant difference (*p* < 0.05); ns: non-significant (*p* > 0.05).

**Table 3:** Muscle fatty acid composition of cultured and wild *C. nasus* (% of total fatty acids).

Fatty Acid (mg/g)	Cultured	Wild	*p* Value
C12:0			
C14:0	2.77 ± 0.11	2.06 ± 0.09	*
C15:0	0.70 ± 0.07	0.30 ± 0.01	*
C16:0	25.23 ± 0.33	27.42 ± 0.38	*
C16:1	7.18 ± 0.25	6.52 ± 0.45	ns
C17:0	0.56 ± 0.08	0.23 ± 0.02	*
C18:0	2.92 ± 0.12	3.43 ± 0.32	ns
C18:1n9t	0.34 ± 0.02	0.27 ± 0.02	*
C18:1n9c	34.47 ± 1.46	39.73 ± 1.56	*
C18:2n6c (LA)	6.32 ± 0.92	0.77 ± 0.12	*
C18:3n3 (ALA)	2.22 ± 0.17	1.10 ± 0.08	*
C20:0	0.68 ± 0.03	0.18 ± 0.02	*
C20:1	1.76 ± 0.18	0.38 ± 0.06	*
C20:2	0.25 ± 0.01	0.14 ± 0.01	*
C20:3n6 (DHLA)	0.56 ± 0.05	-	
C20:4n6 (ARA)	3.56 ± 0.39	1.11 ± 0.24	*
C20:5n3 (EPA)	4.08 ± 0.38	4.45 ± 0.46	ns
C22:1n9	0.24 ± 0.01	0.69 ± 0.10	*
C22:6n3 (DHA)	6.03 ± 0.54	11.02 ± 1.43	*
∑ SFA	32.99 ± 0.34	33.76 ± 0.34	ns
∑ n-3 PUFA	12.33 ± 1.01	16.57 ±1.83	ns
∑ n-6 PUFA	10.44 ± 0.57	1.88 ± 0.34	*
∑ n-3 PUFA/n-6 PUFA	1.21 ± 0.10	10.22 ± 0.50	*

The data are shown as mean ± SEM; * represents significant difference (*p* < 0.05); - represents not detected; ns represents non-significant (*p* > 0.05). LA, linolic acid; ALA, α-linolenic acid; DHLA, dohomo-γ-linolenic acid; ARA, arachidonic acid; EPA, eicosapentaenoic acid; DHA, docosahexaenoic acid; SFA, saturated fatty acid; PUFA, polyunsaturated fatty acid; n-3 PUFA, omega-3 polyunsaturated fatty acid; n-6 PUFA, omega-6 polyunsaturated fatty acid.

## Data Availability

The data, except the sequence data of the gut microbiota and muscle transcriptome, are available from the corresponding author on reasonable request. The sequence data of the gut microbiota and muscle transcriptome that support the findings of this study have been deposited in the NCBI with the primary accession code PRJNA1273278 and PRJNA1273475, respectively.

## Supplementary Materials

The following supporting information can be downloaded at https://www.mdpi.com/article/10.3390/microorganisms13071711/s1, Figure S1: Identification of differentially expressed analysis on muscle transcriptome. A, cluster analysis of expressed genes of 10 libraries. B, principal component analysis (PCA) of expressed genes of 10 libraries. C, volcano plot for muscle gene libraries of the wild and the cultured groups showing the variance in gene expression with respect to fold change (FC) and significance (*p* < 0.05). Wild was set as control group. Cultured was set as treatment group; Figure S2: Gut microbiota composition of *C. nasus* between cultured and wild environments at the phylum and genus levels. A–D, stacked column chart and bar chart representing the composition and significant difference (*p* < 0.05) respectively of phylum and genus whose abundance was in the top 15 between two group; Figure S3: Disparities in alpha and beta diversity of *C. nasus* gut microbiota between cultured and wild environments. A, Calculation of Good’s coverage, Pielou, Shannon and Simpson indexes for *C. nasus*; B, PCoA plot illustrating the gut microbial structure. * represented significant difference (*p* < 0.05); ** represented extremely significant difference (*p* < 0.01).

## Abbreviations

The following abbreviations are used in this manuscript:

ACAA2	Acetyl-CoA Acyltransferase 2
ACOX	Acyl-CoA Oxidase
ACSL4	Acyl-CoA Synthetase Long-Chain Family Member 4
ALA	α-Linolenic Acid
ARA	Arachidonic Acid
ASV	Amplicon Sequence Variant
β2M	Beta-2 Microglobulin
CAT	Catalase
CPT1	Carnitine Palmitoyltransferase 1
CTSB	Cathepsin B
DEG	Differential Gene Expression
DHA	Docosahexaenoic Acid
DHLA	Dohomo-γ-Linolenic Acid
EAAs	Essential Amino Acids
EPA	Eicosapentaenoic Acid
FAD	Flavin Adenine Dinucleotide
FAME	Fatty Acid Methyl Ester
FC	Fold Change
GO	Gene Ontology
GSL	Glycosphingolipids
HMGB1	High Mobility Group Box 1
HSP90	Heat Shock Protein 90
LA	Linolic Acid
LC -PUFAs	Long-chain Polyunsaturated Fatty Acids
LEfSe	Linear Discriminant Analysis Effect Size
LIG3	DNA Ligase III
LPS	Lipopolysaccharides
MCP-1	Monocyte Chemoattractant Protein 1
MDA	Malondialdehyde
MHC	Major Histocompatibility Complex Class
NEAA	Non-Essential Amino Acid
NTH	Endonuclease III-like DNA Glycosylase
PCA	Principal Component Analysis
PCoA	Principal Coordinate Analysis
PPARγ	Peroxisome Proliferator-Activated Receptor Gamma
PUFA	Polyunsaturated Fatty Acid
P53	Tumor Protein P53
RXR	Retinoid X Receptor
SEM	Standard Error
SFA	Saturated Fatty Acids
SOD	Superoxide Dismutase
TAA	Total Amino Acid
T-AOC	Total Antioxidant Capacity
TPA	Texture Profile Analysis
UPGMA	Unweighted Pair-Group Method With Arithmetic Means
WGCNA	Weighted Correlation Network Analysis
WHC	Water-Holding Capacity
