# Supplementary material for: Multi-Omics Analysis Provides New Insights into the Interplay Between Gut Microbiota, Fatty Acid Metabolism, and Immune Response in Cultured and Wild Coilia nasus from the Yangtze River Area in China
Source: Microorganisms. 2025 Jul 21;13(7):1711. doi: 10.3390/microorganisms13071711 (PMC12300613; doi:10.3390/microorganisms13071711)
Supplement: Supplementary file 1 [file microorganisms-13-01711-s001.zip › microorganisms-3724206-supplementary.pdf]

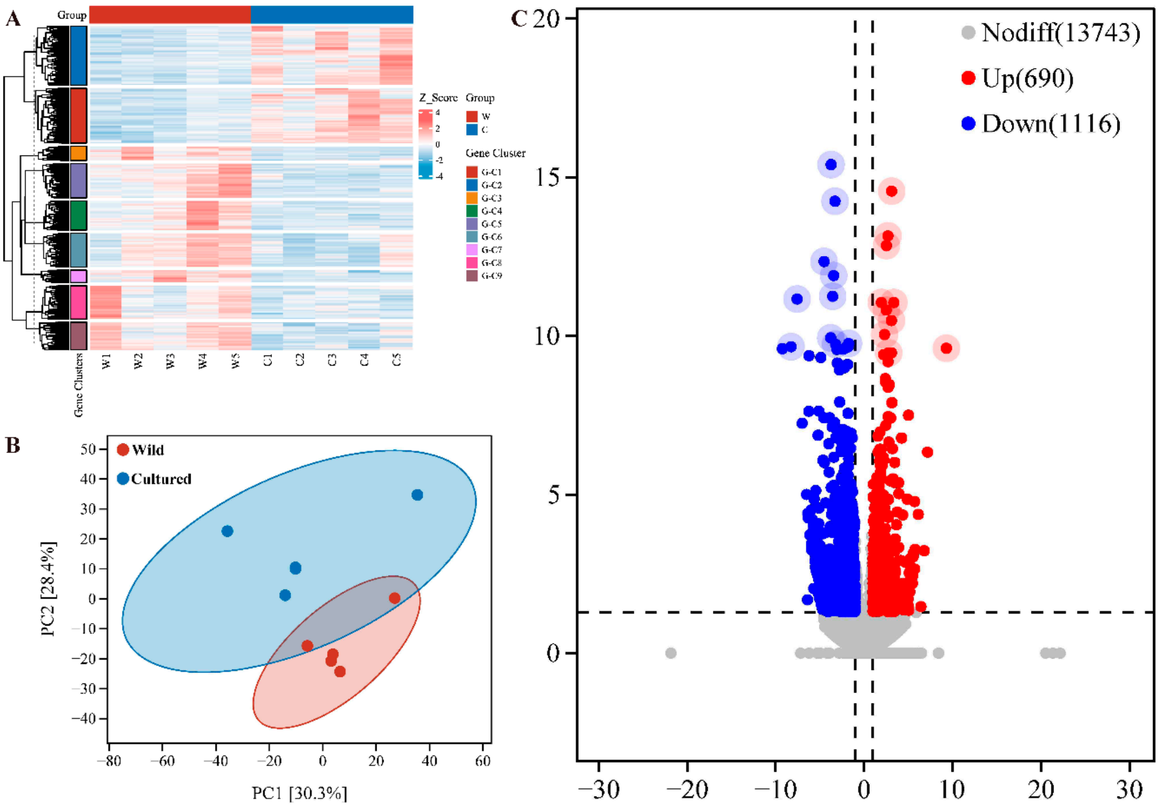

Supplementary Figure S1. Identification of differentially expressed analysis on muscle transcriptome. A, cluster analysis of expressed genes of 10 libraries. B, principal component analysis (PCA) of expressed genes of 10 libraries. C, volcano plot for muscle gene libraries of the wild and the cultured groups showing the variance in gene expression with respect to fold change (FC) and significance (p < 0.05). Wild was set as control group. Cultured was set as treatment group.

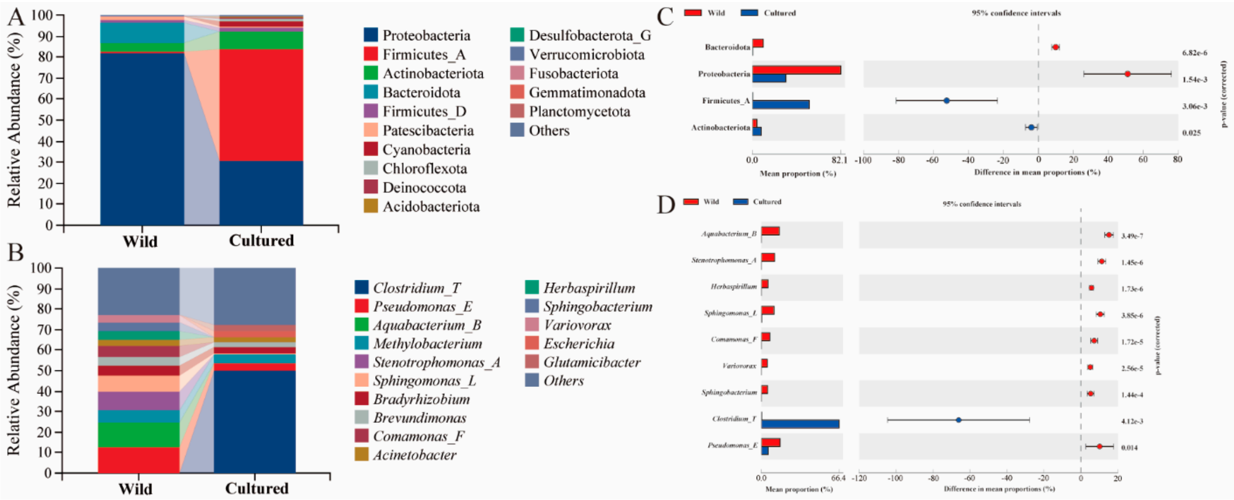

Supplementary Figure S2. Gut microbiota composition of *C. nasus* between cultured and wild environments at the phylum and genus levels. A-D, stacked column chart and bar chart representing the composition and significant difference (p < 0.05) respectively of phylum and genus whose abundance was in the top 15 between two group.

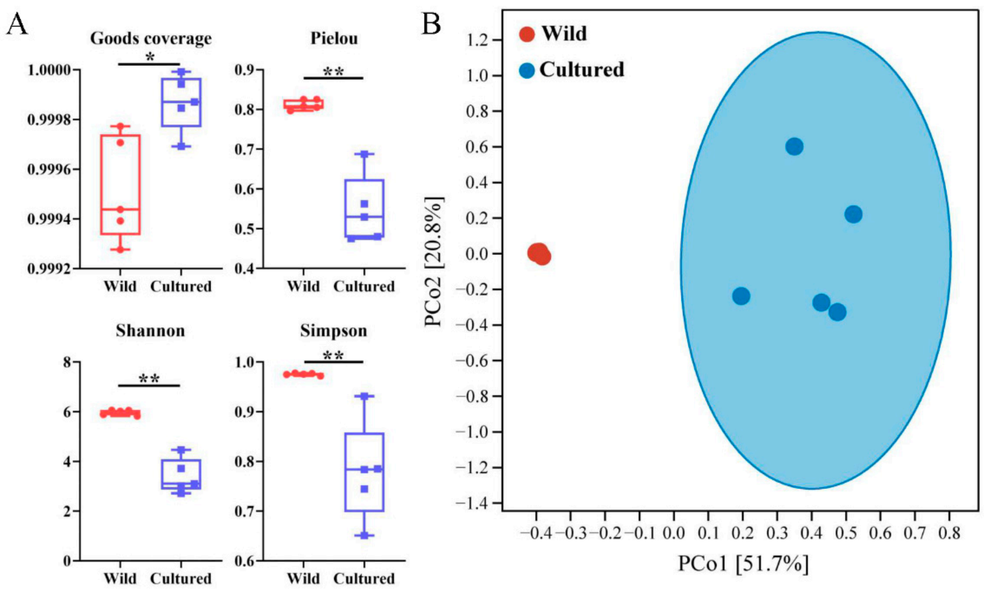

Supplementary Figure S3. Disparities in alpha and beta diversity of *C. nasus* gut microbiota between cultured and wild environments. A, Calculation of Good's coverage, Pielou, Shannon and Simpson indexes for *C. nasus*; B, PCoA plot illustrating the gut microbial structure. * represented significant difference (p < 0.05); ** represented extremely significant difference (p < 0.01).
